# Extinction learning deficit in a rodent model of attention-deficit hyperactivity disorder

**DOI:** 10.1186/1744-9081-8-59

**Published:** 2012-12-13

**Authors:** Ryan J Brackney, Timothy HC Cheung, Katrina Herbst, Jade C Hill, Federico Sanabria

**Affiliations:** 1 Arizona State University, P.O. Box 871104, Tempe, AZ, 85287-1104, USA

**Keywords:** Variable interval, Bout, Extinction, SHR, ADHD, Hierarchical model, Learning, Motivation, Response-outcome association, Dynamic Bi-Exponential Refractory Model (DBERM)

## Abstract

**Background:**

Deficient operant extinction has been hypothesized to be constitutive of ADHD dysfunction. In order to elucidate the behavioral mechanisms underlying this deficit, the performance of an animal model of ADHD, the spontaneously hypertensive rat (SHR), was compared against the performance of a control strain, the Wistar-Kyoto rat (WKY) during extinction.

**Method:**

Following extensive training of lever pressing under variable interval schedules of food reinforcement (reported previously), SHR and WKY rats were exposed to two sessions of extinction training. Extinction data was analyzed using the Dynamic Bi-Exponential Refractory Model (DBERM) of operant performance. DBERM assumes that operant responses are organized in bouts separated by pauses; during extinction, bouts may decline across multiple dimensions, including frequency and length. DBERM parameters were estimated using hierarchical Bayesian modeling.

**Results:**

SHR responded more than WKY during the first extinction session. DBERM parameter estimates revealed that, at the onset of extinction, SHR produced more response bouts than WKY. Over the course of extinction, response bouts progressively shortened for WKY but not for SHR.

**Conclusions:**

Based on prior findings on the sensitivity of DBERM parameters to motivational and schedule manipulations, present data suggests that (1) more frequent response bouts in SHR are likely related to greater incentive motivation, and (2) the persistent length of bouts in SHR are likely related to a slower updating of the response-outcome association. Overall, these findings suggest specific motivational and learning deficits that may explain ADHD-related impairments in operant performance.

## Background

Multiple theories postulate abnormalities in operant conditioning as a behavioral phenotype of attention deficit hyperactivity disorder (ADHD) [1,2]. Some of these theories involve response extinction—the decline in behavior once reinforcement is discontinued—as an aspect of operant performance compromised in ADHD [3,4]. In this regard, theories often make conflicting predictions. Sagvolden and colleagues’ dynamic developmental theory [5], for instance, predicts slower extinction in individuals with ADHD; Tripp and Wickens’ dopamine transfer deficit theory [4] makes the opposite prediction. Empirical evidence that would adjudicate this dispute is surprisingly scarce and difficult to interpret. For instance, Sagvolden and colleagues [5] showed that children with ADHD responded more during extinction than controls; extinction contingencies, however, alternated with positive reinforcement, and schedule interaction effects were not ruled out. Other studies have shown stronger emotional responses to non-reinforcement in children with ADHD [6-8], but very weak differences relative to controls in extinction performance [7]. Research on reversal, omission [9], and Pavlovian extinction learning [10] provide indirect evidence that operant extinction may be slower in individuals with ADHD.

The spontaneously hypertensive rat (SHR), a common animal model of ADHD [11], typically emits higher rates of operant responding under maintenance (when reinforcement is effective and performance is relatively stable) and extinction conditions, relative to the Wistar-Kyoto (WKY) control strain [12,13]. Differences in response rate during extinction, however, only support very limited inferences on extinction deficits, for two reasons. First, response rate during extinction is highly dependent on preceding maintenance response rates [14]. Differences in responding during extinction, therefore, may be due to differences in responsiveness to reinforcement during maintenance, and not due to a fundamental problem with extinction itself [15]. Second, the response rate measure conflates various performance parameters that may be differentially sensitive to extinction contingencies. These parameters arise from the organization of operant behavior in bouts of responses separated by relatively long pauses [16-18].

The Bi-Exponential Refractory Model (BERM) [18,19] of steady-state free-operant maintenance provides estimates of response-bout parameters, including bout initiation rate, response rate within bouts, and average bout length. BERM has been described in detail elsewhere [19]. Briefly, BERM assumes that animals initiate bouts following one Poisson process and, once a bout is initiated, emit responses according to a second, faster, Poisson process. A bout may be exited from with some probability following each response. BERM’s dynamic generalization, DBERM, assumes that a subset of BERM parameters may decline exponentially over time in the absence of reinforcement, such as during extinction. These dynamic components allow for the dissociation of starting parameter estimates (which are dependent on maintenance conditions) from their rate of decline over the course of extinction. This dissociation facilitates the identification of differences between groups, such as the WKY and SHR, in the microstructural dynamics responsible for the decline in response rate during extinction, while minimizing confounds that may arise from unequal starting values.

BERM parameters are differentially sensitive to various experimental manipulations [18,19]. For instance bout initiation rate appears to be particularly sensitive to motivational changes such as food deprivation and reduced reinforcement density. Bout length and within-bout response rate appear to be sensitive primarily to reinforcement contingencies [18], and may thus be indicative of the strength of the response-reinforcer association [1]. BERM and DBERM parameters may, therefore, identify behavioral and cognitive endophenotypes underlying differences in performance between animal models of psychiatric disorders and their controls.

The purpose of the present study was to identify the parameters of operant behavior, as characterized by DBERM, that underlie the observed differences in response rate between SHR and WKY during extinction. Using DBERM allows for the isolation of effects of maintenance contingencies on extinction performance. In addition, strain differences in DBERM parameters may suggest differential sensitivities to contingencies of reinforcement, or differences in the underlying behavioral processes controlling behavior.

## Methods

### Subjects

Twelve male young-adult (PND 94–95) rats, 6 SHR/NCrl (Charles River Laboratories, US) and 6 WKY/NHsd (Harlan Laboratories, US), were used. WKY served as normoactive control for SHR [11]. Rats were food restricted and maintained at approximately 85% of their *ad libitum* weight based on a logistic function fitted to the growth curves provided by breeders. Each rat’s weight was assessed daily, and any negative difference from his expected weight was compensated with an equal weight of food presented an hour after the experimental session terminated. Every rat was fed a minimum of 2 g of post-session food each day, and weighed approximately 240 g at the start of the experiment. Before extinction testing, all subjects participated in a previous study [1], in which additional details on the subjects and apparatus are described. All procedures in the present study were conducted according to the guidelines of the National Institutes of Health, which were approved by the Institutional Animal Care and Use Committee at Arizona State University.

### Apparatus

Experimental sessions were conducted in six MED Associates® operant chambers with the standard dual lever configuration; each retractable lever flanked a food receptacle aperture. Food dispenser activation deposited a single 45-mg food pellet into receptacle. During sessions, only the right lever (nearest the door) was extended.

### Procedure

Prior to extinction testing, all subjects were trained for 54 days on a multiple variable interval (VI) schedule of food reinforcement, operative on a single lever, with schedules ranging between VI 12-s to VI 192-s. The purpose of that training was to assess changes in BERM parameters across multiple schedules of reinforcement in SHR and control strains. The outcome of this assessment is reported in detail elsewhere [1]. Because the multiple-VI training protocol is also described in detail in that report, it is only outlined here. Each session was preceded by a 5-min acclimation period in which no programmed events occurred. Lever access followed the acclimation period for 65 min or 40 reinforcer deliveries, whichever occurred first. A VI schedule was randomly selected without replacement at the beginning of each session from a list of five available schedules (VI 12, 24, 48, 96, or 192-s). An inter-trial interval of 20 s followed each reinforcer (food pellet) delivery, during which the lever was retracted. Each of the five VI schedules was continuously signaled by a pulsing tone of a distinct frequency (3–12 kHz). After eight reinforcers were delivered on a schedule, a new schedule was selected without replacement. Each lever press was followed by a 0.11-s refractory period in which lever pressing was not effective. This refractory period was imposed to prevent the recording of artifactual lever bounces.

Extinction sessions began the day after the final VI training session. Extinction sessions were 65-min long (excluding the preceding 5-min acclimation period), and were identical to VI maintenance sessions in all respects except that only the tone signaling the VI 192-s schedule was sounded and lever pressing had no programmed consequences. Two extinction sessions were conducted over consecutive days.

### Data analysis

Data were analyzed at two levels. At a macrostructural level, the effects of strain (SHR vs. WKY), extinction session (EXT1 vs. EXT2), and time in extinction (eight 8.125-min bins in each session) on log response rate were assessed using a mixed-design ANOVA. Response rates were calculated for each individual rat by dividing the total number of responses within each bin by 8.125 min. ANOVA was conducted in IBM SPSS Statistics® v. 20.

**Figure 1:**
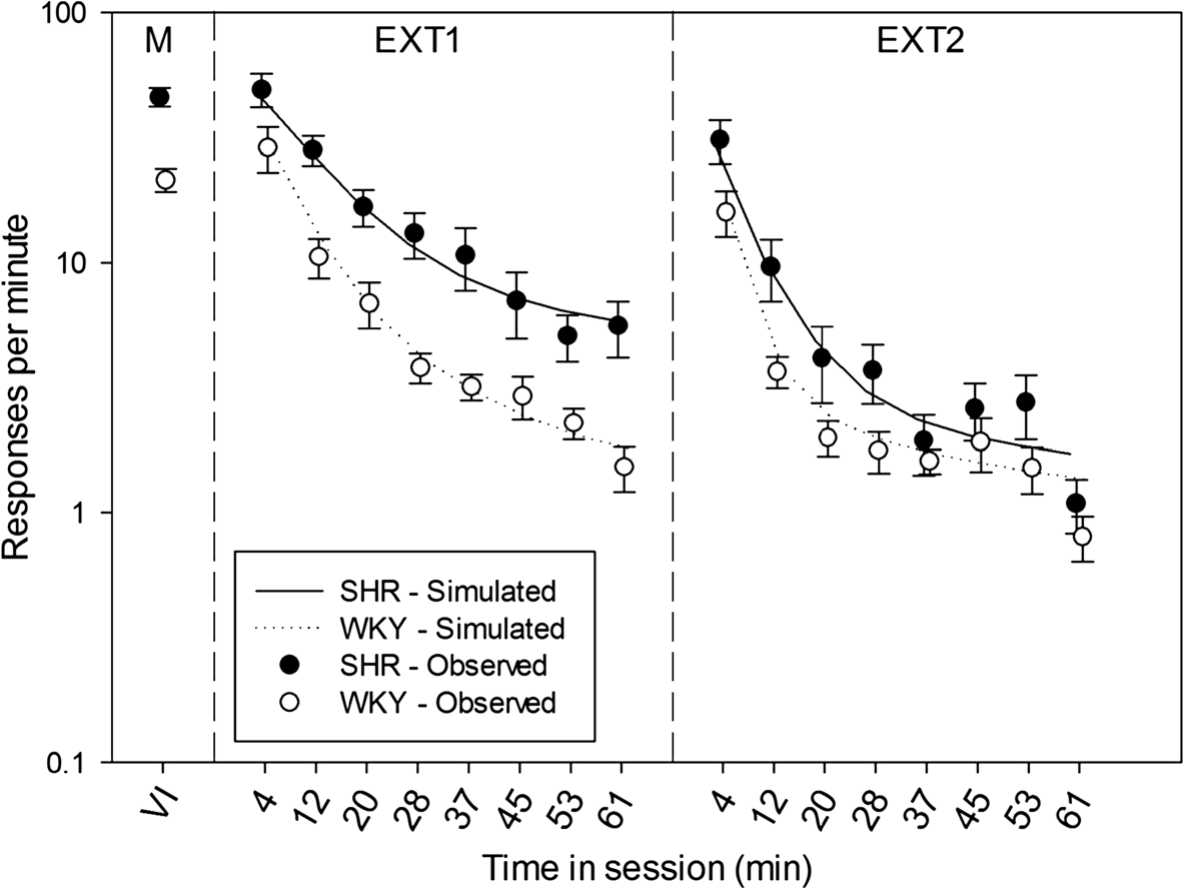
**Mean (+**/**− SEM) response rates during maintenance (M), and extinction sessions (EXT1 and EXT2), in SHR (closed circles) and WKY (open circles), plotted on semi-log coordinates.** Mean simulated rates are shown as curves. Maintenance response rates were calculated from the last four sessions prior to extinction, when the VI 192-s schedule was in effect. Response rates were calculated for each eighth of EXT1 and EXT2 (8.125-min bins; mid-points shown on x-axis). Data points for SHR are slightly shifted to the left for visibility. Response rates declined significantly over the course of EXT1 and EXT2 (p < .004). Response rates were significantly higher for SHR than WKY, but only in EXT1 (p = .008). Mean simulated response rates based on fitted DBERM parameters (Tables 1 and 2) closely tracked the observed response rates during EXT1 and EXT2.

At a microstructural level of analysis, DBERM parameters were estimated from the individual inter-response times (IRTs, the intervals between consecutive lever presses) produced by each rat in each extinction session. Parameter estimation was conducted using a Bayesian hierarchical model in custom written MATLAB® software. More information on the software can be found in [19]. Model details are described immediately below, and in the results section. To verify that DBERM parameters provided a faithful description of performance, predictions of DBERM parameters were plotted against response rates during extinction (Figure 1).

### Model

DBERM was fit to extinction IRTs to identify the sources of ostensible between-strain differences in response rate. DBERM assumes that free-operant performance is described by four separate parameters, three of which may decline as a function of time in extinction *t*. They are the bout initiation rate *b*_*t*_, or rate at which the subject engages in lever-pressing activity; the within-bout response rate *w*_*t*_, or speed at which the lever is activated while engaged; the average bout length *L*_*t*_, which is the mean number of responses in a bout after the response that initiated the bout; and the refractory period *δ*, or minimum time required to emit a response [18-20].

Assuming that responses are independently generated by two Poisson processes with rates *b*_*t*_ and *w*_*t*_, DBERM may be expressed as a mixture of two exponential distributions of IRTs, where the probability that IRT_*t*_ (the IRT that starts at time *t* in extinction) is of duration *τ* is:

(1) $$\begin{array}{l} \mathrm{Pr}\left(IRT_{t}=\tau |\tau <\delta \right)=0\\ \mathrm{Pr}\left(IRT_{t}=\tau |\tau \geq \delta \right)=p_{t}w_{t}e^{-wt\left(\tau -\delta \right)}+\left(1-p_{t}\right)b_{t}e^{-b_{t}\left(\tau -\delta \right)}\;w_{t}>b_{t}. \end{array}$$

Parameter *p*_*t*_, the probability of remaining in bout at time *t*, may be computed from the average bout length *L*_*t*_[19]:

(2) $$p_{t}=\frac{L_{t}}{1+L_{t}.}$$

To account for changes in response rate during extinction, DBERM allows *L*_*t*_, *w*_*t*_, and *b*_*t*_ to decline exponentially over the course of extinction, starting at *L*_*0*_, *w*_*0*_, and *b*_*0*_. These last three parameters will henceforth be referred to as *baseline parameters*, and their derived estimates at each time *t* as *dynamic parameters*:

(3) $$\begin{array}{ll} L_{t}=L_{0}e^{-\gamma t} & L_{0},\gamma \geq 0\\ w_{t}=\left(w_{0}-\Omega \right)e^{-\alpha t}+\Omega & w_{0}>b_{0}\geq \Omega \geq 0\\ b_{t}=\left(b_{0}-\Omega \right)e^{-\beta t}+\Omega & \beta \geq \alpha \geq 0. \end{array}$$

Parameters *γ*, *α*, and *β* are the decay rates of *L*_*t*_, *w*_*t*_, and *b*_*t*_, respectively. These decay rates may be expressed as the half-life of the corresponding parameter, for ease of interpretation [e.g., *H*_*b*_ = ln(2) / *β*. Parameters *w*_*t*_ and *b*_*t*_ are assumed to asymptote to rate Ω, which may be the operant level of the response [21]. Baseline parameters and their half-lives are listed in Table 1. Whereas baseline parameters (*L*_*0*_, *w*_*0*_, and *b*_*0*_) in the first extinction session (EXT1) are dependent on maintenance conditions, the half-lives of these parameters (*H*_*L*_, *H*_*w*_, and *H*_*b*_) are not necessarily so. Thus, the comparison between the half-lives of DBERM parameters of SHR and WKY may identify the processes responsible for the differences in the decline in response rate during extinction, while minimizing potential confounds that arise from differences during maintenance.

**Table 1:** Estimated DBERM parameter medians (95% credible interval) for each strain in EXT1

**Parameter**	** Description**	**SHR**	**WKY**
*Baseline Parameters*			
*L_0_*	Mean bout length^†^ (resp)	0.91 (0.31-2.61)	2.81 (1.79 – 4.27)
*w_0_*	Within-bout response rate (min^-1^)	253.71 (90.76 – 687.57)	175.68 (99.49 – 307.82)
*b_0_*	Bout-initiation rate (min^-1^)	42.74 (27.54 -70.92)*	14.70 (8.76 – 25.14)*
*Half-Lives*			
*H_L_*	Half-life of *L* (min)	2794.48 (79.50 – 6.9 × 10^5^)*	19.37 (9.35 – 55.72)*
*H_w_*	Half-life of *w* (min)	42.94 (9.77 – 244.32)	21.28 (5.80 – 104.20)
*H_b_*	Half-life of *b* (min)	7.77 (4.81 – 12.88)	4.69 (1.58 – 11.26)
*Ancillary Parameters*			
δ	Refractory period (s)	0.11 (0.11 – 0.11)	0.12 (0.10 – 0.15)
Ω	Asymptotic response rate (min^-1^)	2.37 (0.81 – 6.76)	1.64 (1.07 – 2.50)

^†^Bout length is the number of responses in a bout minus the bout-initiating response. E.g., a bout of length 1 means that 2 responses comprised the bout, whereas a bout length 0.5 means that *on average*, one within-bout response occurs for every two bout-initiation responses. This arrangement is designed to allow bout length to decline to zero over extinction, as indicated in Equation 3.

*Significant difference between strains (95% credible interval of differences between strains did not envelop zero).

In order to account for potential within-subject changes in DBERM parameters between EXT1 and EXT2, the following model was used: For each subject, let *x*_1_ be a placeholder for a DBERM parameter on EXT1 (e.g., *L*_*0*_), and *x*_2_ be a placeholder for the same parameter on EXT2. It was assumed that.

(4) $${Χ}_{2}=C\left(x\right)\cdot x_{1}$$

where *C*(*x*) denotes the *recovery coefficient* for parameter *x*. For example, if the baseline bout length on EXT2 was half of that on EXT1, then *C*(*L*_*0*_) would be 0.5. This model therefore assumes a multiplicative change of parameters between sessions, analogous to the within-session exponential decay assumed by DBERM. If a parameter did not change between sessions, the estimate of its recovery coefficient would be close to 1.

There were a total of 16 model parameters for each rat: 8 DBERM parameters describing performance on EXT1 (Table 1), and 8 recovery coefficients describing performance on EXT2 (Table 2). Parameters were estimated using Bayesian hierarchical modeling [19,22-25]. This approach imposed a hierarchical structure to account for data variability. Each rat’s IRT data were assumed to be distributed as described by Equations 1, 2, 3 and 4 according to the rat’s own *individual* DBERM parameters. Each individual parameter was assumed to be log-normally distributed among rats within each strain group. Finally, flat (uniform) prior distributions were assumed for the mean (μ) and standard deviation of the log-normal distributions of individual parameters due to the limited information about group parameters before the experiment.

**Table 2:** Estimated recovery coefficient medians (95% credible interval) for each strain in EXT2

**Parameter**	**SHR**	**WKY**
*C*(*L*_*0*_)	1.44 (0.89 – 2.41)*	0.50 (0.25 – 1.04)*
*C*(*w*_*0*_)	0.85 (0.55 – 1.34)	0.93 (0.76 – 1.14)
*C*(*b*_*0*_)	0.73 (0.55 – 0.94)	1.24 (0.51 – 3.41)
C(*H*_*L*_)	0.15 (0.00 – 128.70)	1.27 (0.46 – 9.47)
*C*(*H*_*w*_)	0.52 (0.18 – 1.48)	1.51 (0.77 – 6.65)
*C*(*H*_*b*_)	0.44 (0.22 – 0.89)	0.43 (0.15 – 1.05)
*C*(δ)	1.00 (0.98 – 1.00)	1.09 (0.85 – 1.36)
*C*(Ω)	0.37 (0.26 – 0.58)*	0.70 (0.49 – 1.02)*

*Significant difference between strains (95% credible interval of differences between strains did not envelop zero).

For a given DBERM parameter *x*, the measure of interest was the mean of the posterior distribution of each strain, μ_*x*(SHR)_ and μ_*x*(WKY)_. Inferences about the differences between strains were based on the posterior distributions of the (unstandardized) effect size, *E*_*x*_ = μ_*x*(SHR)_ – μ_*x*(WKY)_. The mean and the central 95% credible interval (CI) of each of these posterior distributions were estimated using Markov chain Monte Carlo (MCMC) [22,26,27]. The 95% CI contains 95% of the mass of the posterior distribution. A significant difference between the strains was declared for model parameter *x* if the 95% CI of *E*_*x*_ did not include 0. The method for MCMC sampling is described in detail elsewhere [19]. A total of 20100 MCMC samples of the joint posterior distribution were collected.

In order to aid interpretation, the posterior estimates of the log-normal group mean parameters, effect sizes, and their 95% CIs were back-transformed to the linear scale using exponentiation. The back-transformed μ_*x*_ is an estimate of the median DBERM parameter *x* for a strain on the linear scale. The linear effect size is an estimate of the ratio of the (linear) group medians of the two strains, and a significant difference implies that the 95% CI of this ratio does not include 1.

## Results

### Response rate

Mean (+/− SEM) response rates during maintenance and extinction sessions 1 and 2 (EXT1 and EXT2) are shown as dots in Figure 1. Each extinction session was divided into eight bins of 8.125 min. A 2 × 2 × 8 (strain: SHR vs. WKY, session: EXT1 vs. EXT2, bin: 1 vs. 2 vs. … 8) mixed-design ANOVA was conducted with log response rate as the dependent measure. A significant main effect of strain was detected, F(1,10) = 6.93, p = 0.025, as well as a significant main effect of session, F(1, 10) = 93.57, p < 0.001. A strain × session interaction effect was also detected, F(1, 10) = 5.82, p = 0.037. Simple main effects tests revealed that SHR responded substantially more than WKY during EXT1, F(1, 10) = 10.79, p = 0.008, but not during EXT2, F(1,10) = 2.40, p = 0.152.

A significant main effect of bin, F(7, 70) = 71.52, p < 0.001, and a session × bin interaction, F(7,70) = 2.36, p = 0.032, were also observed. A pairwise comparison of response rates in bin 1 across sessions found that initial responding in EXT1 was significantly greater (p = 0.004) than in EXT2. In EXT1, pairwise comparisons between consecutive bins indicated that response rate in each bin was significantly higher (p ≤ 0.05) than the response rate in the next bin for all but bins 5 vs. 6. In contrast, pairwise comparisons of consecutive bins in EXT2 revealed a significant difference (p ≤ 0.05) of response rate only between bins 1 vs. 2, 2 vs. 3, and 7 vs. 8, suggesting that response rate had reached near asymptotic levels after the third bin. There were no significant strain × bin or strain × bin × session interaction effects on log response rate. Combined, these results indicate that (1) SHR responded substantially more than WKY in EXT1, (2) this difference between strains subsided by EXT2, (3) regardless of strain, responding at the start of the session was substantially faster in EXT1 than EXT2, and (4) response rate decayed to near asymptotic levels substantially faster in EXT2 than in EXT1.

### DBERM parameters

DBERM was fit to 19,184 individual IRTs collected from all animals in both EXT sessions. Parameter estimates for individual subjects may be found in the supplementary material - Additional file 1: Tables S1 and S2. Table 1 summarizes the posterior distributions of parameters for EXT1, on the linear scale. The estimate of the SHR median *b*_*0*_ was almost 3 times larger than that of WKY, indicating that, at the onset of extinction, SHR produced substantially more response bouts than WKY. The half-lives of *L*_*t*_, *w*_*t*_, and *b*_*t*_ for EXT1 are also included in Table 1. Only *H*_*L*_ varied significantly between strains: the median bout length of SHR was virtually constant during extinction (*H*_*L*_ = 2794.48 min)^a^, whereas WKY’s declined to half of its baseline estimate within 20 min. It is possible that SHR’s long *H*_*L*_ was due to the group’s low *L*_*0*_. That is, SHR could have emitted too few within-bout responses for the parameters associated with the within-bout state to be estimated accurately. However, a simulation experiment (described in section 2 of the supplementary material) demonstrated that even with a lower *L*_*0*_, *H*_*L*_ can be estimated with reasonable accuracy, suggesting that the lack of evidence for SHR’s *L*_*0*_ declining during the session, and the differences in *H*_*L*_ between SHR and WKY, were not statistical artifacts.

Parameter estimates for EXT2 are conveyed as recovery coefficients (Equation 4) in Table 2. Significant between-strain differences were observed only in *C*(*L*_*0*_) and *C*(Ω). These results indicate that (1) bout length at the onset of extinction declined less between sessions for SHR than for WKY, and (2) asymptotic response rate appeared to decline more between sessions for SHR than for WKY.

The goodness of fit of DBERM was validated using posterior predictive check. Ten thousand samples of the joint posterior distribution of DBERM parameters of individual rats were randomly selected without replacement from the MCMC output. For each sample, the following Monte Carlo simulation of EXT1 and EXT2 was conducted. For each rat, an IRT starting at session time *t*_*n*_ (IRT_*n*_, where *n* is a response counter) was generated using the following algorithm: (1) Update the rat’s dynamic parameters (*L*_*t*_, *w*_*t*_, *b*_*t*_) at *t*_*n*_. This was accomplished by substituting *t*_*n*_ and the sampled DBERM parameters for the rat into Equation 3. Sampled recovery coefficients were also used to compute DBERM parameters for EXT2. (2) Randomly sample the bout state at *t*_*n*_. The probability of being in the within-bout state was *p*_*t*_; the probability of being in the between-bout state was 1 – *p*_*t*_. (3) Randomly sample a pause of duration *τ*_*n*_ from an exponential distribution with mean pause of either 1/*w*_*t*_ or 1/*b*_*t*_ depending on the sampled bout state. (4) Add *δ* to the sample of *τ*_*n*_ to give IRT_*n*_. After generating IRT_*n*_, the session timer was increased to *t*_*n*+1_ = *t*_*n*_ + IRT_*n*_, and IRT_*n*+1_ was generated by repeating from step 1 with *t*_*n*+1_. This iterative procedure was carried out starting from *t*_1_ = 0, for each rat, for each of the two extinction sessions, until the session time *t* exceeded the experimental session time of 65 min. This generated a prediction of how every rat would respond conditional on the observed experimental data and the model constraints imposed by DBERM. The simulated overall response rates for the SHR and WKY groups, averaged over the 10000 samples of the posterior distribution, are reported in Figure 1 as curves. Simulations closely tracked the changes in response rate observed during both extinction sessions, thus validating DBERM and the extinction parameter estimates in Tables 1 and 2 as reasonable descriptions of extinction in SHR and WKY.

**Figure 2:**
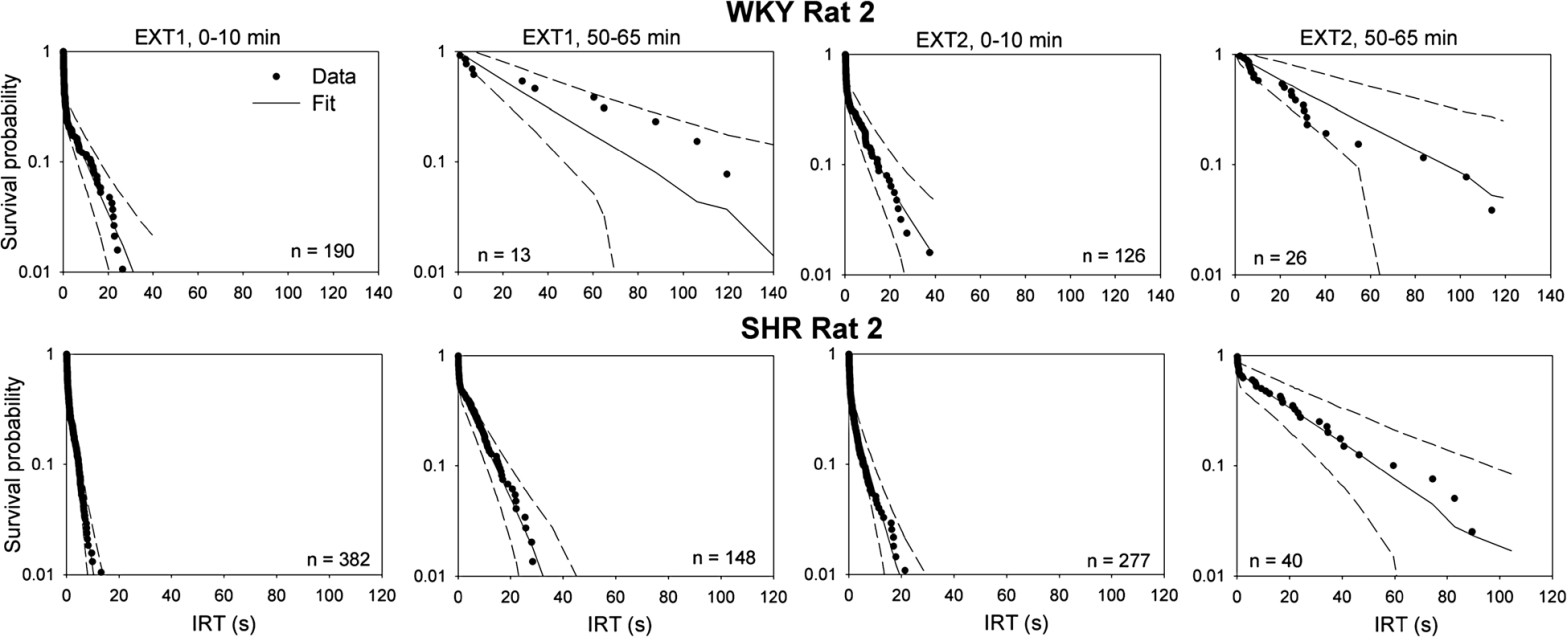
**Log survival plots of IRTs from 4 different extinction periods from a representative rat of each group.** Dots show data; solid and broken lines show the median and central 95 percentile of predicted log survival probabilities as generated using Monte Carlo simulation based on samples of the posterior estimates of DBERM parameters. The number of observed IRTs contained in each period is also shown. Similar plots for remaining individuals in each group are shown in Additional file 1: Figures S1 and S2 in the supplementary materials.

In addition, the performance of DBERM on fitting each rat’s IRT distribution was assessed using the same posterior predictive check. For simplicity, we confined our check to the IRT distributions in 4 periods: the first 10 min in EXT1, the last 15 min in EXT1 (50–65 min), the first 10 min in EXT2, and the last 15 min in EXT2. The IRT distribution for each period from each rat was plotted as log survival plots, whose “broken-stick” shape first suggested to investigators that responses were generated by two different processes [20]. The log survival plots for a representative rat from each group are shown as dots in Figure 2. Plots for the remaining rats are shown in the supplementary material - Additional file 1: Figures S1 and S2. Note that plots from the end of the extinction sessions have fewer IRTs. It is evident that the shape of the plots and their change within and across extinction sessions is complex. For example, the shallower slope of the right-hand tail of the function as extinction progressed provides a visual indication that the rate of bout initiation was declining during extinction. The shortening of the left-hand limb of the function also suggests that the average bout length was shortening during extinction. The median posterior prediction (solid lines in Figure 2; Additional file 1: Figure S1 and S2) suggests that DBERM does a reasonable job at fitting the complex dynamics of IRTs with little systematic bias. The variances of the posterior prediction are in most cases small as shown by narrow 95% credible intervals around the medians, which suggest that DBERM did not overfit the data. Noticeable deviations were confined to cases with few data points (e.g., end of EXT2 for SHR Rat 5 and WKY Rat 6), and the vast majority of IRTs were well described by DBERM.

A previous paper by Cheung et al. [19] references the current data set for illustrative purposes. Differences in data analysis between this paper and [19] are discussed in supplemental material section 4.

## Discussion

This study was aimed at identifying the aspects of operant responding that are responsible for the difference in extinction performance between SHR and WKY. As this study and others [17-19] indicate, free operant responding is composed of multiple underlying components that change over the course of extinction. These changes may reflect the numerous processes implicated in extinction learning, such as reductions in arousal or incentive motivation [28-30], and learning new associations in an unreinforced context [31,32], among many others (see [31,33] for reviews). A precise mapping of these processes to changes in response-bout microstructure during extinction [18] would provide a foundation for inferences about the processes responsible for differences in performance between strains.

In EXT1 of the current study, SHR responded more than WKY, which is consistent with prior studies that compared the extinction performance of these strains [3,34,35]. DBERM parameter estimation revealed that SHR emitted more response bouts at the onset of extinction. Because deprivation level and reinforcement density covary positively with bout initiation rate [16,18,20,36-39] the difference in mean *b*_*0*_ between strains suggests a heightened motivation for incentives in SHR. This heightened motivation has been proposed as a fundamental component of operant hyperactivity in SHR [1]. Effects on *b*_*0*_ are likely carried over from differences in maintenance performance, which means that the difference in *b*_*0*_ between strains is not unique to extinction performance. During extinction the context of reinforcement incites responding [28,40], presumably influencing *b*. In this study, we observed no significant difference in *H*_*b*_ and *C*(*b*_0_) between strains, which also suggests that differences in extinction performance between SHR and WKY cannot be explained by differences in the decline of context-elicited arousal [40].

We also observed significant difference of *C*(Ω) between strains. The steeper reduction of Ω in SHR between extinction sessions undermines the notion that Ω is simply the operant level of the response. Parameter Ω and its change between sessions may depend on unknown factors that decline at a slow rate over extinction, such as the occasion-setting properties of the context [41]. Further investigation into the appropriate estimation and the theoretical interpretation of Ω is required before sound behavioral inferences based on Ω can be made.

Of the theoretical perspectives that specifically address response extinction, the present findings are most consistent with Sagvolden’s dynamic development theory of ADHD [5]. This theory predicts slower extinction in individuals with ADHD compared to neurotypical controls. The dynamic development theory assumes that individuals with ADHD have shortened delay of reinforcement gradients. A shorter delay of reinforcement gradient would attenuate the length of response sequences that are reinforced [2], which would be reflected in shorter initial bout lengths (*L*_*0*_). While a significant difference of *L*_*0*_ was not observed in EXT1, we did detect a trend in *L*_*0*_ consistent with Sagvolden’s theory and with estimates from maintenance performance [1].

DBERM parameter estimation also demonstrated that bout length decayed over the course of extinction for WKY, but not for SHR. Bout length is particularly sensitive to schedule manipulations even when reinforcement rate remains constant [18,39,42]. Such sensitivity suggests that bout length is an indicator of the strength of the response-outcome association [1,18]. During extinction, the response-outcome association is expected to weaken (even if it is not completely obliterated [32]), a process that may be expressed as a progressive reduction in bout length. Therefore, it is likely that, after operant conditioning on VI schedules, the absence of decay in bout length in SHR during extinction is indicative of a slower updating of the response-reinforcer association. This slower process may reflect, in turn, a fundamental learning deficit. The strain difference in the between-session change of *L*_*0*_ suggests that the slow associative updating process attributed to SHR within EXT1 also operated between extinction sessions.

An alternative explanation of the constant bout length observed in SHR during extinction may attribute such effect to the low baseline bout length of SHR. That is, SHR bouts may have declined very little in length, relative to WKY bouts, because SHR bouts were already very short, and not because of any learning deficit in SHR. This explanation is unlikely to be valid for two reasons, besides the statistical non-significance in the difference of *L*_0_ between strains. First, median DBERM parameter estimates indicate that WKY bouts declined in length at such rate that SHR and WKY bouts were of equal length around the middle of the EXT1 (more precisely, after 31.73 min). That is, the same parameter estimates from which the constant bout length of SHR is inferred suggest that WKY bouts were shorter than SHR bouts during the second half of EXT1. If SHR bouts were too close to the minimal bout length, then the model would have underestimated WKY bout length and response rate during the second half of EXT1, a divergence that is not observed in the simulation (cf. Figure 1). Second, we simulated extinction performance using individual SHR parameters, except that *H*_*L*_ was sampled from WKY estimates (described in supplemental material, section 2). When parameters were recovered from the simulation, we found no evidence of bias: the credible intervals of the recovered parameters enveloped the parameters that produced the simulated data. We did not find evidence of bias even when the generative SHR *L*_0_ was shortened by a factor of 3. This absence of bias suggests that the DBERM was sensitive enough that, had bout length in SHR declined at a rate comparable to that of WKY, DBERM would have detected it.

It may seem surprising that the SHR median within-bout response rate is estimated at over 250 resp/min. Note however that the minimum time between responses (δ) has been observed to be on the order of 0.1 s across multiple experiments [18,19], indicating a “top speed” of 600 resp/min, although this rate is not typically sustained over long intervals due to the pause-and-bout pattern of responding. In addition, within-bout response rates exclude δ (cf. Equation 1). With these considerations, it can be found that the currently observed within-bout response rates are well within a plausible range.

Potential issues regarding data interpretation may also arise from the multiple-VI training that preceded the current experiment. It is unlikely that the particular order in which VI schedules were presented in the last maintenance session influenced extinction performance, because such order was randomized in every multiple-VI session. However, it is possible that the current results are idiosyncratic to extinction following multiple (as opposed to simple) VI training. To assess the generality of our findings, similar comparisons of SHR and WKY during extinction should be conducted following training with a single VI.

It should also be noted that DBERM is a descriptive model. Currently it has 8 parameters describing dynamic responding within an extinction session (Table 1). Previous work using Akaike information criterion with model selection suggests that all 8 parameters describing within-session responding are required [19]. Instead of having a new set of 8 DBERM parameters for each extinction session, we used recovery coefficients for each DBERM parameters as a way to describe their changes across multiple extinction sessions (Table 2). The recovery coefficients assumed that DBERM parameters change multiplicatively across extinction sessions. Further experiments will be needed to examine how well multiplicative recovery coefficients describe extinction data beyond the first two extinction sessions.

## Conclusion

At the beginning of extinction SHR emitted more response bouts (*b*_*0*_) whose length declined at a slower rate *H*_*L*_ and *C*(*L*_0_)]. This implies that SHR emitted more responses than WKY following the discontinuation of operant reinforcement. The difference in baseline bout-initiation rate most likely reflects differences in maintenance performance carried over to extinction, and not differences in extinction learning itself. In contrast, the persistent length of bouts in SHR suggests deficient extinction learning in SHR. Because changes in bout length are primarily related to schedule effects [18,20,39,42], it is likely that the persistent length of SHR bouts reflects a low sensitivity to changes in reinforcement schedule. This inference is consistent with findings of slower autoshaping in adult SHR [43], and with the slow responsiveness of individuals with ADHD to changes in reinforcement contingencies [9,10,44]. Thus, the identification of extinction learning deficits in SHR supports its use as an animal model of ADHD-related learning deficits, which may be involved in some varieties of hyperactivity [1]. In general terms, this evidence provides further support for the use of SHR as an animal model of ADHD [11,45].

## Endnote

^a^ Estimates of *H*_*L*_ were capped at 5.6 × 10^6^ min (approximately 10 years). Half lives are capped because of the possibility of infinite half-lives, which can lead to the posterior distributions of the decay rate parameters (γ, α, β) to be improper, i.e., the posterior distribution integrates to infinity [22].

## Abbreviations

ADHD: Attention deficit hyperactivity disorder; BERM: Bi-exponential refractory model; DBERM: Dynamic bi-exponential refractory model; EXT1 and EXT2: Extinction sessions 1 and 2; SHR: Spontaneously hypertensive rat strain; WKY: Wistar-Kyoto rat strain; VI: Variable interval schedule.

## Competing interests

The authors declare they have no competing interests.

## Authors’ contributions

JH and KH collected the data. RB and TC performed the data analysis. TC designed the algorithms used to conduct the hierarchical Bayesian analysis. RB drafted the manuscript with assistance from TC and FS. FS conceived of the study and provided general oversight. All authors read and approved the final manuscript.

## Supplementary Material

Additional file 1 **Individual subject parameter estimates, additional simulation results and explanations, and comparisons of data in **[19]. Click here for file

## References

[B1] Hill JC Herbst K Sanabria F Characterizing Operant Hyperactivity in the Spontaneously Hypertensive Rat Behav Brain Funct 2012 8 5 10.1186/1744-9081-8-5 22277367 PMC3292830

[B2] Luman M Tripp G Scheres A Identifying the neurobiology of altered reinforcement sensitivity in ADHD: a review and research agenda Neurosci Biobehav R 2010 34 744 754 10.1016/j.neubiorev.2009.11.021 19944715

[B3] Johansen EB Aase H Meyer A Sagvolden T Attention-deficit/hyperactivity disorder (ADHD) behaviour explained by dysfunctioning reinforcement and extinction processes Behav Brain Res 2002 130 37 45 10.1016/S0166-4328(01)00434-X 11864716

[B4] Tripp G Wickens JR Research review: dopamine transfer deficit: a neurobiological theory of altered reinforcement mechanisms in ADHD J Child Psychol Psyc 2008 49 691 704 10.1111/j.1469-7610.2007.01851.x 18081766

[B5] Sagvolden T Johansen EB Aase H Russell VA A dynamic developmental theory of attention-deficit/hyperactivity disorder (ADHD) predominantly hyperactive/impulsive and combined subtypes Behav Brain Sc 2005 28 397 419 discussion 419–68 16209748 10.1017/S0140525X05000075

[B6] Douglas VI Parry PA Effects of reward and nonreward on frustration and attention in attention deficit disorder J Abnorm Child Psych 1994 22 281 302 10.1007/BF02168075 8064034

[B7] Iaboni F Douglas VI Ditto B Psychophysiological response of ADHD children to reward and extinction Psychophysiology 1997 34 116 123 10.1111/j.1469-8986.1997.tb02422.x 9009815

[B8] Wigal T Swanson JM Douglas VI Wigal SB Wippler CM Cavoto KF Effect of reinforcement on facial responsivity and persistence in children with attention-deficit hyperactivity disorder Behav Modif 1998 22 143 10.1177/01454455980222003 9563288

[B9] Itami S Uno H Orbitofrontal cortex dysfunction in attention-deficit hyperactivity disorder revealed by reversal and extinction tasks Neuroreport 2002 13 2453 10.1097/00001756-200212200-00016 12499848

[B10] Frings M Gaertner K Buderath P Gerwig M Christiansen H Schoch B Gizewski ER Hebebrand J Timmann D Timing of conditioned eyeblink responses is impaired in children with attention-deficit/hyperactivity disorder Exp Brain Res 2010 201 167 176 10.1007/s00221-009-2020-1 19777220

[B11] Sagvolden T Johansen EB Wøien G Walaas SI Storm-Mathisen J Bergersen LH Hvalby O Jensen V Aase H Russell VA Killeen PR Dasbanerjee T Middleton FA Faraone SV The spontaneously hypertensive rat model of ADHD--the importance of selecting the appropriate reference strain Neuropharmacology 2009 57 619 626 10.1016/j.neuropharm.2009.08.004 19698722 PMC2783904

[B12] Sagvolden T Hendley ED Knardahl S Behavior of hypertensive and hyperactive rat strains: Hyperactivity is not unitarily determined Physiol Behav 1992 52 49 57 10.1016/0031-9384(92)90432-2 1529013

[B13] Johansen EB Sagvolden T Behavioral effects of intra-cranial self-stimulation in an animal model of attention-deficit/hyperactivity disorder (ADHD) Behav Brain Res 2005 162 32 46 10.1016/j.bbr.2005.02.033 15922065

[B14] Guttman N Operant conditioning, extinction, and periodic reinforcement in relation to concentration of sucrose used as reinforcing agent J Exp Psychol 1953 46 213 13109117 10.1037/h0061893

[B15] Alsop B Problems with spontaneously hypertensive rats (SHR) as a model of attention-deficit/hyperactivity disorder (AD/HD) J Neurosci Meth 2007 162 42 48 10.1016/j.jneumeth.2006.12.002 17241669

[B16] Podlesnik CA Jimenez-Gomez C Ward RD Shahan TA Resistance to change of responding maintained by unsignaled delays to reinforcement: A response-bout analysis J Exp Anal Behav 2006 85 329 10.1901/jeab.2006.47-05 16776055 PMC1459851

[B17] Shull RL Gaynor ST Grimes JA Response rate viewed as engagement bouts: resistance to extinction J Exp Anal Behav 2002 77 211 10.1901/jeab.2002.77-211 12083677 PMC1284858

[B18] Brackney RJ Cheung THC Neisewander JL Sanabria F The isolation of motivational, motoric, and schedule effects on operant performance: A modeling approach J Exp Anal Behav 2011 96 17 38 10.1901/jeab.2011.96-17 21765544 PMC3136892

[B19] Cheung THC Neisewander JL Sanabria F Extinction under a behavioral microscope: isolating the sources of decline in operant response rate Behav Process 2012 90 111 123 10.1016/j.beproc.2012.02.012 PMC3336036 22425782

[B20] Shull RL Gaynor ST Grimes JA Response rate viewed as engagement bouts: effects of relative reinforcement and schedule type J Exp Anal Behav 2001 75 247 10.1901/jeab.2001.75-247 11453618 PMC1284817

[B21] Catania AC The operant reserve: a computer simulation in (accelerated) real time Behav Process 2005 69 257 278 10.1016/j.beproc.2005.02.009 15845312

[B22] Gelman A Bayesian data analysis 2004 London: CRC press

[B23] Griffiths TL Kemp C Tenenbaum JB Cleeremans A, Dienes Z Bayesian models of cognition Cambridge handbook of computational cognitive modeling 2008 New York: Cambridge University Press 59 100

[B24] Shiffrin RM Lee MD Kim W Wagenmakers EJ A survey of model evaluation approaches with a tutorial on hierarchical Bayesian methods Cognitive Sci 2008 32 1248 1284 10.1080/03640210802414826 21585453

[B25] Rouder JN Lu J An introduction to Bayesian hierarchical models with an application in the theory of signal detection Psychon B Rev 2005 12 573 604 10.3758/BF03196750 16447374

[B26] MacKay DJC Information theory, inference, and learning algorithms 2003 Cambridge, UK: Cambridge University Press

[B27] Robert CP Casella G Monte Carlo statistical methods 1999 New York: Springer-Verlag

[B28] Podlesnik CA Sanabria F Repeated extinction and reversal learning of an approach response supports an arousal-mediated learning model Behav Process 2011 87 125 134 10.1016/j.beproc.2010.12.005 PMC4762371 21172410

[B29] Killeen PR Mathematical Principles of Reinforcement Behav Brain Sc 1994 17 105 172 10.1017/S0140525X00033628

[B30] Bindra D How adaptive behavior is produced: a perceptual-motivational alternative to response reinforcements Behav Brain Sc 1978 1 41 52 10.1017/S0140525X00059380

[B31] Bouton ME Context and behavioral processes in extinction Learn Memory 2004 11 485 494 10.1101/lm.78804 15466298

[B32] Rescorla R Inhibitory associations between S and R in extinction Learn Behav 1993 21 327 336 10.3758/BF03197998

[B33] Lattal KM Lattal KA Facets of Pavlovian and operant extinction Behav Process 2012 90 1 8 10.1016/j.beproc.2012.03.009 PMC3337697 22465468

[B34] Johansen EB Sagvolden T Response disinhibition may be explained as an extinction deficit in an animal model of attention-deficit/hyperactivity disorder (ADHD) Behav Brain Res 2004 149 183 196 10.1016/S0166-4328(03)00229-8 15129781

[B35] Johansen EB Sagvolden T Slower extinction of responses maintained by intra-cranial self-stimulation (ICSS) in an animal model of attention-deficit/hyperactivity disorder (ADHD) Behav Brain Res 2005 162 22 31 10.1016/j.bbr.2005.02.035 15922064

[B36] Reed P An experimental analysis of steady-state response rate components on variable ratio and variable interval schedules of reinforcement J Exp Psychol Anim B 2011 37 1 9 10.1037/a0019387 20718555

[B37] Johnson JE Pesek EF Newland MC High-rate operant behavior in two mouse strains: a response-bout analysis Behav Process 2009 81 309 315 10.1016/j.beproc.2009.02.013 19429225

[B38] Conover KL Fulton S Shizgal P Operant tempo varies with reinforcement rate: implications for measurement of reward efficacy Behav Process 2001 56 85 101 10.1016/S0376-6357(01)00190-5 11672935

[B39] Shull RL Bouts of responding on variable-interval schedules: effects of deprivation level J Exp Anal Behav 2004 81 155 10.1901/jeab.2004.81-155 15239490 PMC1284977

[B40] Killeen PR Wynne CDL, Staddon JER The First Principle of Reinforcement Models of action: Mechanisms for adaptive behavior 1998 Hillsdale: Lawrence Erlbaum 127

[B41] Ross RT Holland PC Conditioning of simultaneous and serial feature-positive discriminations Learn Behav 1981 9 293 303 10.3758/BF03197835

[B42] Shull RL Grimes JA Bouts of responding from variable-interval reinforcement of lever pressing by rats J Exp Anal Behav 2003 80 159 10.1901/jeab.2003.80-159 14674726 PMC1284951

[B43] Meneses A Castillo C Ibarra M Hong E Effects of aging and hypertension on learning, memory, and activity in rats Physiol Behav 1996 60 341 345 8840889

[B44] Kollins SH Lane SD Shapiro SK Experimental analysis of childhood psychopathology: A laboratory matching analysis of the behavior of children diagnosed with attention-deficit hyperactivity disorder (ADHD) Psychol Rec 1997 47 25 44

[B45] Sanabria F Killeen PR Evidence for impulsivity in the Spontaneously Hypertensive Rat drawn from complementary response-withholding tasks Behav Brain Funct 2008 4 7 10.1186/1744-9081-4-7 18261220 PMC2276225

