# Supplementary material for: Extinction learning deficit in a rodent model of attention-deficit hyperactivity disorder
Source: Behav Brain Funct. 2012 Dec 13;8:59. doi: 10.1186/1744-9081-8-59 (PMC3542014; doi:10.1186/1744-9081-8-59)
Supplement: Additional file 1 — Individual subject parameter estimates, additional simulation results and explanations, and comparisons of data in [19]. [file 1744-9081-8-59-S1.docx]

**Supplementary materials**

1. Posterior estimates of individual parameters

Table S1. Units of DBERM parameters

| **Parameter** | **Unit** |
| --- | --- |
| *L_0_* | resp |
| *w_0_* | min^-1^ |
| *b_0_* | min^-1^ |
| *H_L_* | min |
| *H_w_* | min |
| *H_b_* | min |
| *δ* | s |
| *Ω* | min^-1^ |

Table S2. Individual parameters for the 6 rats in the WKY group. Posterior means were back-transformed from the log scale to the linear scale. Therefore, the numbers shown are the (back-transformed) posterior medians (numbers in brackets are the 95% credible interval).

|  | **WKY 1** | **WKY 2** | **WKY 3** | **WKY 4** | **WKY 5** | **WKY 6** |
| --- | --- | --- | --- | --- | --- | --- |
| *L_0_* | 2.73  (1.80-4.11) | 2.63  (1.80-3.94) | 2.58  (1.89-3.48) | 1.95  (1.23-3.08) | 4.13  (3.11-5.52) | 3.35  (2.57-4.47) |
| *w_0_* | 77.49  (60.28-98.82) | 243.41  (181.70-324.61) | 189.25  (158.05-227.44) | 166.84  (121.27-228.94) | 226.15  (199.00-256.23) | 222.91  (190.44-262.03) |
| *b_0_* | 13.95  (6.54-43.01) | 10.94  (7.36-16.59) | 17.61  (12.40-25.40) | 13.47  (7.69-22.68) | 21.77  (15.21-31.36) | 12.60  (9.18-17.78) |
| *H_L_* | 41.99  (14.52-637.87) | 14.11  (7.27-30.72) | 24.62  (16.70-42.32) | 16.14  (7.85-35.05) | 16.19  (12.25-22.12) | 13.87  (10.24-19.48) |
| *H_w_* | 9.00  (7.27-11.80) | 8.72  (6.03-15.84) | 131.66  (41.53-2284.12) | 14.45  (9.03-50.16) | 29.66  (20.74-45.90) | 21.39  (14.35-38.70) |
| *H_b_* | 2.04  (0.37-9.21) | 5.65  (2.95-9.91) | 5.00  (3.31-7.34) | 3.33  (1.84-6.24) | 5.62  (3.84-8.18) | 10.00  (5.66-16.57) |
| *δ* | 0.13  (0.11-0.14) | 0.17  (0.16-0.17) | 0.11  (0.11-0.11) | 0.12  (0.11-0.12) | 0.11  (0.11-0.11) | 0.11  (0.11-0.11) |
| *Ω* | 2.64  (1.75-3.46) | 1.52  (1.06-2.04) | 1.28  (0.93-1.71) | 1.30  (0.95-1.70) | 1.63  (1.16-2.15) | 1.80  (1.10-2.62) |
| C(*L_0_*) | 0.40  (0.17-2.32) | 0.46  (0.27-0.81) | 0.76  (0.46-1.38) | 0.33  (0.16-0.62) | 0.79  (0.52-1.23) | 0.44  (0.28-0.70) |
| C(*w_0_*) | 0.95  (0.75-1.25) | 0.96  (0.75-1.31) | 0.91  (0.71-1.12) | 0.91  (0.64-1.23) | 0.93  (0.78-1.11) | 0.91  (0.70-1.12) |
| C(*b_0_*) | 3.43  (0.92-9.20) | 1.29  (0.73-2.29) | 1.12  (0.41-3.65) | 1.02  (0.53-1.96) | 1.00  (0.51-1.92) | 0.71  (0.40-1.26) |
| C(*H_L_*) | 0.87  (0.03-13.63) | 1.40  (0.02-2.83) | 1.25  (0.59-2.80) | 1.10  (0.01-1.42) | 0.99  (0.56-1.40) | 0.98  (0.57-1.66) |
| C(*H_w_*) | 0.72  (0.38-1.21) | 0.67  (0.14-1.51) | 1.24  (0.03-1.74) | 0.47  (0.10-1.22) | 0.90  (0.54-1.65) | 0.98  (0.07-1.15) |
| C(*H_b_*) | 0.74  (0.59-12.70) | 0.74  (0.83-3.88) | 0.56  (1.59-17.68) | 0.56  (0.64-2.83) | 0.92  (1.37-4.97) | 0.53  (0.91-4.54) |
| C(*δ*) | 2.63  (0.72-1.06) | 1.83  (1.32-1.47) | 4.94  (1.15-1.30) | 1.36  (0.88-1.24) | 2.61  (0.96-1.01) | 2.03  (0.89-1.02) |
| C(*Ω*) | 0.63  (0.44-0.89) | 0.85  (0.58-1.39) | 0.63  (0.41-0.90) | 0.67  (0.46-0.96) | 0.79  (0.57-1.18) | 0.69  (0.45-1.08) |

Table S3. Individual parameters for the 6 rats in the SHR group. Posterior means were back-transformed from the log scale to the linear scale. Therefore, the numbers shown are the (back-transformed) posterior medians (numbers in brackets are the 95% credible interval).

|  | **SHR 1** | **SHR 2** | **SHR 3** | **SHR 4** | **SHR 5** | **SHR 6** |
| --- | --- | --- | --- | --- | --- | --- |
| *L_0_* | 0.47  (0.36-0.65) | 0.98  (0.82-1.22) | 1.74  (1.33-2.44) | 2.72  (1.49-5.17) | 0.29  (0.19-0.43) | 0.96  (0.83-1.13) |
| *w_0_* | 351.24  (258.53-480.14) | 297.99  (256.22-351.87) | 125.33  (101.66-154.88) | 95.74  (83.43-110.95) | 240.07  (140.26-382.09) | 898.68  (769.83-1047.51) |
| *b_0_* | 34.16  (26.74-44.23) | 27.92  (23.53-33.46) | 39.80  (32.19-48.62) | 63.81  (47.22-83.32) | 43.25  (37.36-49.92) | 67.41  (57.98-78.29) |
| *H_L_* | 4017.92  (42.39-2.41×10^6^) | 1626.86  (81.77-6.49×10^5^) | 2001.86  (70.41-7.94×10^5^) | 4249.27  (62.11-1.94×10^6^) | 5400.15  (60.07-2.16×10^6^) | 1653.46  (87.11-5.86×10^5^) |
| *H_w_* | 38.50  (21.57-93.57) | 317.87  (87.03-7723.90) | 45.44  (30.08-78.45) | 15.21  (12.43-19.47) | 18.25  (10.16-46.38) | 39.42  (28.54-59.16) |
| *H_b_* | 5.47  (3.93-7.47) | 14.42  (9.05-22.18) | 8.18  (6.53-10.30) | 5.82  (4.30-7.50) | 8.04  (7.08-9.11) | 7.28  (6.03-8.78) |
| *δ* | 0.11  (0.11-0.11) | 0.11  (0.11-0.11) | 0.11  (0.11-0.11) | 0.11  (0.11-0.11) | 0.11  (0.11-0.11) | 0.11  (0.11-0.11) |
| *Ω* | 3.21  (2.51-3.97) | 5.25  (2.65-7.71) | 2.12  (1.38-3.03) | 2.03  (1.32-2.88) | 0.56  (0.22-1.06) | 4.34  (3.26-5.45) |
| C(*L_0_*) | 1.51  (1.03-2.19) | 1.49  (1.04-2.12) | 1.56  (0.94-2.72) | 1.41  (0.72-2.67) | 1.96  (1.19-3.34) | 0.91  (0.66-1.26) |
| C(*w_0_*) | 0.62  (0.35-0.97) | 0.75  (0.59-0.96) | 1.10  (0.77-1.57) | 0.70  (0.55-0.88) | 1.16  (0.70-2.24) | 0.91  (0.69-1.22) |
| C(*b_0_*) | 0.61  (0.38-0.84) | 0.73  (0.55-0.94) | 0.79  (0.58-1.12) | 0.76  (0.53-1.11) | 0.71  (0.56-0.88) | 0.78  (0.64-0.97) |
| C(*H_L_*) | 0.44  (0.00-731.64) | 47.93  (2.03-1.87×10^4^) | 39.52  (0.02-2.69×10^4^) | 1.62  (0.00-2152.52) | 0.79  (0.00-1093.19) | 70.39  (3.16-2.48×10^4^) |
| C(*H_w_*) | 1.55  (0.73-3.74) | 1.82  (0.22-10.00) | 4.35  (1.99-9.00) | 0.82  (0.58-1.14) | 2.01  (1.06-5.18) | 2.65  (1.52-4.58) |
| C(*H_b_*) | 1.82  (1.13-2.80) | 2.49  (1.57-3.94) | 2.76  (2.05-3.75) | 5.08  (3.40-7.50) | 1.84  (1.53-2.21) | 1.14  (0.91-1.43) |
| C(*δ*) | 1.00  (0.97-1.00) | 1.00  (0.98-1.00) | 1.00  (0.98-1.00) | 1.00  (0.97-1.00) | 1.00  (0.98-1.04) | 1.00  (0.99-1.00) |
| C(*Ω*) | 0.36  (0.26-0.50) | 0.40  (0.27-0.76) | 0.33  (0.20-0.49) | 0.35  (0.23-0.54) | 0.43  (0.26-1.20) | 0.36  (0.24-0.51) |

1. Posterior estimates of individual parameters

It is possible that, with the low *L_0_* of the SHR group, there were very few within-bout responses and as a result, the parameters associated with the within-bout state cannot be estimated with high levels of precision. Note that the converse can also be true – with a very high L_0_ there can be too few bout initiations for the parameters associated with the bout-initiation state to be estimated accurately.

We tested whether the low *L_0_* of SHR was able to detect a faster decrease in *L_t_* (i.e., higher *γ* and thus lower *H_L_*) with reasonable precision and no bias by conducting the following Monte Carlo experiment. The posterior estimates of the DBERM parameters for individual subjects from EXT1 were retrieved. The *γ* of the 6 SHR rats was substituted with the *γ* of the 6 WKY rats. Which SHR rat had the *γ* of which WKY rat was randomly assigned without replacement. Thus, this new simulated group of 6 rats had WKY’s fast *γ* (and thus short H_L_) and SHR’s low *L_0_* (and all other SHR rats’ parameters). Then, a Monte Carlo simulation of IRTs on EXT1 was generated for each rat using the same method as the posterior predictive check simulation outlined in the paper. The same Bayesian hierarchical analysis used in the paper was used to estimate this new group’s DBERM parameters. The resultant back-transformed group median estimates were compared to SHR’s EXT1 group medians, except for *H_L_*, which was compared to WKY’s EXT1 group median. Results in Table S4 show that (1) there was no bias in any of the group median estimates, evidenced by the 95% credible intervals of the simulated medians covering the original medians, (2) despite the low *L_0_*, the Bayesian analysis was able to estimate *H_L_* with reasonable precision (width of 95% CI) relative to the original estimate for WKY (whose 95% CI is 9.35 – 55.72, see Table 1 in paper).

Table S4. Short *H_L_* can be detected despite low *L_0_*. Numbers in parenthesis are the 95% credible interval. Original medians are from Table 1 in the paper.

| *Parameter* | *Original group median* | *Median estimate of simulation* |
| --- | --- | --- |
| *L_0_* | 0.91 (SHR) | 1.17 (0.30 – 4.93) |
| *w_0_* | 253.71 (SHR) | 268.40 (88.36 – 812.92) |
| *b_0_* | 43.74 (SHR) | 40.10 (26.18 – 62.10) |
| ***H_L_*** | **19.37 (WKY)** | **14.35 (7.57 – 27.88)** |
| *H_w_* | 42.94 (SHR) | 50.72 (16.27 – 830.51) |
| *H_b_* | 7.77 (SHR) | 8.63 (5.34 – 14.50) |
| *δ* | 0.11 (SHR) | 0.11 (0.11 – 0.11) |
| *Ω* | 2.37 | 1.44 (0.10 – 8.99) |

We further tested whether an even lower *L_0_* would affect the precision with which *H­_L_* could be estimated. We took the same 6 simulated rats as above (with posterior means of SHR rats except for *H_L_* which was from WKY rats). We lowered each rat’s *L_0_* by multiplying them by *e*^-1^ (0.36). The Monte Carlo IRT simulation and Bayesian hierarchical analysis was repeated. The reference group median for L_0_, originally from the SHR group, was also multiplied by 0.36 to reflect the change. The resultant “original median” for *L_0_* was thus lowered to 0.33, i.e., one within-bout response for every three bout initiation responses. Results in Table S5 show that (1) there was no bias in any of the estimates of group medians, (2) estimate of *H_L_* still has reasonable precision. This shows that estimate of *H_L_* and its precision are relatively unaffected by very low *L_0_*, suggesting that the long *H_L_* estimate of the SHR group in the paper was not due to *L_0_* being too low. Interestingly, a very low *L_0_* reduced the precision *H_w_* estimates. This is because there were very few within-bout responses to begin with, and because bout length declined early in the session, there was almost no within-bout response data late in the session for *H_w_* to be accurately estimated, and therefore the data were consistent with a wide range of *H_w_* values. Note however that despite this lack of precision, there was no bias in the estimate of *H_w_,* and that the 95% covered the original median.

Table S5. Low *H_L_* can be detected despite very low *L_0_*. Numbers in parenthesis are the 95% credible interval. Original medians are from Table 1 in paper except that for *L_0_*.

| *Parameter* | *Original group median* | *Median estimate of simulation* |
| --- | --- | --- |
| ***L_0_*** | **0.33 (Lowered)** | **0.31 (0.14 – 0.61)** |
| *w_0_* | 253.71 (SHR) | 265.36 (112.21 – 597.18) |
| *b_0_* | 43.74 (SHR) | 45.97 (29.05 – 72.44) |
| ***H_L_*** | **19.37 (WKY)** | **13.75 (5.53 – 42.54)** |
| *H_w_* | 42.94 (SHR) | 895.49 (16.17 – 9.18×10^5^) |
| *H_b_* | 7.77 (SHR) | 7.40 (4.59 – 12.43) |
| *δ* | 0.11 (SHR) | 0.11 (0.11 – 0.11) |
| *Ω* | 2.37 (SHR) | 2.32 (0.50 – 8.84) |

1. Log survivor plots of IRTs.





Figure S1. Log survivor plots of IRTs from 4 different extinction periods from individual WKY rats. Dots show data, while the predicted log survival probabilities as generated using Monte Carlo simulation using samples of the posterior estimates of DBERM parameters are shown as solid line (median) and broken lines (central 95 percentile). The number of observed IRTs contained in each period is also shown.





Figure S2. Log survivor plots of IRTs from 4 different extinction periods from individual SHR rats. Dots show data, while the predicted log survival probabilities as generated using Monte Carlo simulation using samples of the posterior estimates of DBERM parameters are shown as solid line (median) and broken lines (central 95 percentile). The number of observed IRTs contained in each period is also shown.

4. Current data analysis compared to Cheung et al [19].

A previous paper by Cheung et al [19] references the current data set for illustrative purposes. The results presented there are the same, except that [19] only examined EXT1, the lower bounds for parameter estimates were set to *e*^-15^ for [19] and *e*^-20^ for the current paper. Despite the difference in lower bounds, parameter estimate of EXT1 agreed between the two analyses – all of the median DBERM parameters of EXT1 in this paper lie within the 95% CI of [19] (see their Table 4), and vice versa. Both papers also found the same effect of strain on EXT1. However, the median estimates (and their 95% CI) are not *identical* because (1) lower bounds are different, and (2) the stochastic nature of MCMC means that parameter estimates will differ slightly between analyses on the same data set even with the exact same priors and model. The magnitude of this difference will decrease to zero as the number of MCMC samples used tends to infinity.
